# Respiratory Syncytial Virus: A Narrative Review of Updates and Recent Advances in Epidemiology, Pathogenesis, Diagnosis, Management and Prevention

**DOI:** 10.3390/jcm14113880

**Published:** 2025-05-30

**Authors:** Ali Alsuheel Asseri

**Affiliations:** Department of Child Health, College of Medicine, King Khalid University, Abha 62529, Saudi Arabia; alsoheel11@kku.edu.sa; Tel.: +966-500186013

**Keywords:** RSV, pathogenesis, acute lower respiratory infection, bronchiolitis, monoclonal antibodies, maternal vaccination

## Abstract

Respiratory syncytial virus (RSV) continues as the major cause of acute lower respiratory tract infections in children around the world, and its substantial morbidity, particularly among infants and high-risk children, poses a significant burden on healthcare systems worldwide. RSV infections occur as a spectrum, ranging from mild upper respiratory symptoms to severe bronchiolitis and pneumonia, and the number of infections shows seasonal variations in different latitudes, as well as lasting impacts, reflecting the COVID-19 pandemic. The pathogenesis of the virus involves epithelial cell invasion and/or fusion to form syncytia, along with exaggerated immune-mediated responses. Disease severity is known to depend on viral load, strain variation, and host immune immaturity. Severe RSV infection during infancy is notably linked with long-term respiratory sequelae such as recurrent wheezing and asthma. Diagnosis is based on clinical suspicion and laboratory confirmation using rapid antigen testing or nucleic acid amplification tests, namely PCR. Non-pharmaceutical interventions, maternal vaccination, and prophylaxis with monoclonal antibodies, e.g., palivizumab and nirsevimab, a newly introduced long-acting agent, are efficient protective and preventive measures. Treatment is still, for the most part, supportive in nature and focuses on oxygen supplementation, hydration, and respiratory support for patients with more severe disease courses; however, the development of immunoprophylaxis and vaccine candidates shows promise for reducing the global burden of RSV.

## 1. Introduction

Respiratory syncytial virus (RSV) is a leading cause of acute lower respiratory tract infections (LRTIs) in children worldwide and also a major cause of pediatric morbidity and mortality [1,2]. Specifically, it is globally reported to be responsible for about 33 million LRTIs annually, resulting in about 3.6 million hospitalizations and nearly 100,000 deaths among children less than five years old, especially in low- and middle-income countries (LMICs) [2,3]. The substantial burden is on young infants under six months of age and high-risk populations such as preterm infants or infants with known cardiopulmonary disease or immunodeficiency status [4,5,6]. All told, RSV infections are considered a substantial source of healthcare and economic burden, as they lead to the need for hospitalization and intensive care and, hence, to significant use of health systems worldwide [7,8].

RSV infection presents with a range of clinical manifestations, from mild upper respiratory tract symptoms to severe bronchiolitis and pneumonia requiring supportive care; in severe cases, mechanical ventilation is recommended [9]. It has been concluded that disease clinical phenotype is affected by viral load, strain variability (RSV-A and RSV-B), and host-related factors, including age, immune immaturity, and genetic predisposition [2,10,11,12,13,14,15,16,17,18,19]. Incomplete immunity to RSV results in repeated infections and, thereby, causes a heavy disease burden, including recurrent wheezing and possible asthma [10]. RSV shows strong seasonal epidemiologic patterns, occurring during the winter in temperate regions and during the rainy season in tropical climates [11,12]. However, prolonged public health measures such as mask wearing, isolation, and school closures have proven to distort this seasonality [2,13].

RSV pathogenesis is complex, consisting of epithelial cell invasion and viral replication followed by an exaggerated immune-mediated inflammatory reaction that results in airway obstruction and severe disease [14,15,16]. The management of RSV infection predominately employs supportive care, including oxygen therapy and hydration. Due to cost and dosing constraints, prophylaxis with palivizumab (Synagis™–AstraZeneca) is applied only in high-risk infants [20,21]. However, promising advances in RSV prevention and management have recently been reported. Maternal vaccines directed against serotypes that commonly affect infants are effective at providing passive immunity to infants [6,22], and the long-acting monoclonal antibody nirsevimab (Beyfortus, Sanofi, Paris, France), which neutralizes the two serotypes of RSV (A and B), has also demonstrated efficacy in clinical trials [6,23].

In light of the importance of RSV and related research developments, this narrative review summarizes the recent epidemiology, pathogenesis, clinical presentation, diagnosis, treatment, prevention, and long-term consequences of RSV infection in children. It also includes some results of global studies and emphasizes the effects of the COVID-19 pandemic on RSV epidemiological and clinical patterns.

## 2. Methods

### 2.1. Search Strategy

To obtain a complete synthesis of the literature, the search was carried out in multiple electronic databases, including PubMed, Web of Science, and Scopus, and included all forms of publications from 1956 to 2025. The search was conducted using the keywords “Respiratory Syncytial Virus”, “epidemiology”, “pathogenesis”, “treatment”, “prevention”, and associated terms. To narrow the search, only peer-reviewed articles written in English with human studies were included. Additional filters on article type, study design, and relevance were applied to refine the results to the most pertinent studies.

### 2.2. Selection Criteria

The inclusion criteria were specifically designed to capture a wide array of studies pertinent to the research objectives. These criteria comprised original data, including epidemiology, clinical presentations, management, pathogenesis, and prevention, disseminated in a peer-reviewed article.

### 2.3. Exclusion Criteria

Articles published in non-peer-reviewed journals outside the 1956–2025 timeframe, not written in English, and not involving human subjects were excluded. In an effort to keep our focus on empirical evidence, we also excluded editorials, commentaries, and opinions.

## 3. History of RSV

RSV was first identified in 1956 when researchers isolated a virus from chimpanzees with upper respiratory manifestations. This virus was originally called the ‘chimpanzee coryza agent’ before being renamed RSV because of the syncytial cytopathic effect it produces in infected cells, forming multinucleated cells [24]. Shortly after, RSV was determined to be a major cause of respiratory infections in humans, especially infants and young children.

RSV was a focus of vaccine research in the 1960s. Early trials used a formalin-inactivated vaccine, but this vaccine resulted in partial immunity and unexpectedly led to enhanced respiratory disease during natural infections in vaccinated children [25], a phenomenon that impeded further vaccine development and demonstrated the intricacy of RSV immunity [26]. In the 1990s, advancements in antibody-based therapies resulted in the approval of palivizumab for disease prevention in high-risk infants. While palivizumab reduces RSV-related hospitalizations, its high cost has led to it being used only in very-high-risk infants [27]. The recent approval of nirsevimab, a long-acting monoclonal antibody requiring only a single intramuscular injection for season-long protection, represents a significant advance in RSV immunoprophylaxis [28].

**Figure 1:**
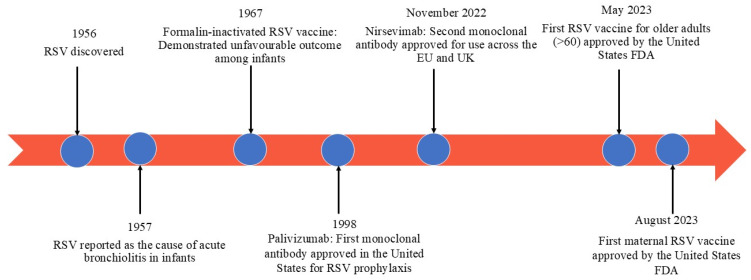
Timeline of key developments in respiratory syncytial virus (RSV) research and prevention.

Vaccine technology has also advanced in recent years, spurring action in the prevention of RSV. The first RSV vaccines, approved in 2023 and developed by Arexvy (GSK) and Abrysvo (Pfizer), target both older adults and infants through maternal immunization and thereby reduce morbidities and mortalities associated with RSV across many age groups. Figure 1 provides a summarized timeline of the major developments in RSV research and preventative strategies since its discovery [20].

## 4. Epidemiology

### 4.1. Global and Regional Prevalence

RSV remains a significant and leading cause of mortality and morbidity in children worldwide, especially children younger than 5 years. A recent report estimated that the global burden of RSV is approximately 33 million LRTIs per year, with 3.6 million hospital admissions and nearly 100,000 deaths in children < 5 years old [2,29]. Although most RSV-related deaths occur as a result of the disease burden, over 90% were seen in LMICs, where access to adjunctive medical care and prophylactic measures is limited [20,30]. The burden of RSV infectivity is significant in Saudi Arabia, with high infant hospitalization rates, as modelled by Alharbi et al. in 2024 [23]. The estimated RSV-related resource utilization is also substantial, resulting in a call for developing immunoprophylaxis programmes in the Kingdom [22,31].

Even in developed regions such as North America and Europe, RSV continues to be the most common cause of pediatric hospitalizations [2,32]. In the United States, 57–72% of bronchiolitis hospitalizations in infants younger than one year are attributed to RSV, with the highest incidence occurring among those between 2 and 6 months of age [2,3,33]. European data, likewise, suggest that RSV is the most common cause of hospitalization in infants, with a reported annual incidence rate of 20–40 cases per 1000 infants under one year old [3,32,34].

Additionally, several region-specific studies, particularly in tropical and sub-Saharan regions, have revealed a significant health burden from RSV infections. For instance, African countries report serious RSV hospitalization rates of over 40 per 1000 infants infected annually, disproportionately high mortality rates in cases of delayed diagnosis, and limited access to healthcare [4,35]. Likewise, in Southeast Asia, morbidity due to RSV spikes during seasonal outbreaks has been associated with environmental factors that enhance RSV transmission and severity, such as humidity and overcrowding [10,36,37].

Overall, while global epidemiological data about RSV are available, data from LMICs are still scarce. Given that most RSV epidemiological data from LMICs are hospital based [2,4,37], these results, therefore, need to be interpreted with caution.

### 4.2. Seasonal Variations

The timing and regularity of RSV activity vary by region, dictated by local climatologic and environmental factors. In temperate climates, RSV cases exhibit seasonal patterns, peaking in November to February in the Northern Hemisphere and May to August in the Southern Hemisphere [38,39]. However, this pattern can probably be attributed to seasonally higher levels of indoor crowding, lower levels of ventilation, and lower indoor temperatures, which would allow the virus to survive and spread. In Saudi Arabia, RSV cases peak during cooler months, a seasonality similar to temperate climates [22,23]. Meanwhile, in tropical areas, RSV waves occur during the rainy season, with frequent indoor confinement and humid conditions again promoting viral transmission. For example, studies in sub-Saharan Africa and Southeast Asia have shown RSV surges aligning with the months with the highest rainfall [20,40].

Traditional RSV seasonality was markedly perturbed during the COVID-19 pandemic; in particular, during 2020 and early 2021, the number of RSV cases decreased globally as a result of the implementation of widespread non-pharmaceutical interventions, such as masking, school closures, social distancing, and travel restrictions [11]. For example, in Australia, the number of RSV infections during winter 2020 decreased by 98 percent compared to previous years [41]. However, when restrictions were lifted, RSV returned with atypical epidemiology and different seasonality patterns [42,43]. These delayed seasonal peaks and intensified outbreaks sometimes amount to unprecedented summer RSV outbreaks in Australia, the US, and parts of Europe [38,41]. These data can provide a window into the intricate interrelationship between viral transmission dynamics and control strategies.

More recently, longitudinal data including four winter seasons from the Vancouver metropolitan region (BC, Canada) revealed a significant trend in female healthcare and school workers of childbearing age; following a resurgence of RSV in their metropolitan region after a period of low circulation due to pandemic measures, key indicators of RSV immunity, including viral neutralization titers, pre-F-specific IgM and IgG levels, and antibody-dependent cellular phagocytosis, gradually returned to pre-pandemic levels within two winter seasons. Notably, pre-F IgG avidity remained stable throughout the study period. These temporal dynamics suggest that repeated exposure to the virus, and subsequent reinfections, plays a crucial role in maintaining robust and functional RSV antibody levels within the adult population [44].

### 4.3. Risk Factors for Severe RSV Infection

Demographic, clinical, environmental, and genetic factors play specific roles in the severity of RSV infection.

1. Age: While RSV can infect any age group, infants younger than 6 months old are at the highest risk of severe disease since their immune systems are not yet fully developed and their airways are smaller. Neonates, especially those that are premature, are most vulnerable to severe RSV infection [9]. Recently, several studies have reported that older adults (≥60 years) develop severe RSV, particularly those with cardiopulmonary comorbidity [36]. The proposed mechanisms of severe RSV among older adults include altered innate and adaptive immune response to viral infections.
2. Prematurity and other conditions: Preterm infants with RSV are 2–3-times more likely to be hospitalized to the critical care [43]. Premature infants, especially those of gestational age < 35 weeks, have compromised surfactant production due to the underdevelopment of their lungs; in addition, maternal antibody transfer is less efficient. RSV outcomes, e.g., prolonged hospital stays and mortality, are also significantly elevated in children with congenital heart disease, chronic lung diseases such as bronchopulmonary dysplasia, or immunodeficiencies [20,45].
3. Environmental factors: RSV disease severity is further exacerbated by environmental contributors, especially in LMICs. The following factors have known associations with RSV severity:
  - Exposure to tobacco smoke resulting in impaired airway inflammation and ciliary function [46].
  - Crowding in living conditions and daycare facilities, which promotes viral transmission [47].
4. Viral load: The association between RSV viral load and the disease severity of RSV is not well understood. El Saleeby et al. reported a significant correlation between RSV viral load and the duration of hospitalization, as well as the need for intensive care and mechanical ventilation [48]. Furthermore, in another study, after adjusting for potential confounders, including age, gestational age, sex, comorbidity, RSV subgroup, and the interval between symptom onset and sample acquisition, infants presenting with febrile RSV infection exhibited a significantly higher peak viral load compared to those with afebrile RSV infection (7.1 [SD 1.2] vs. 6.6 [SD 1.4] log10 copies/mL; *p* = 0.042) [49]. In contrast, another study did not show any association between viral load and RSV disease severity [50]. These contrasting results of association between RSV viral load and disease severity are likely due to the lack of tools that clearly assess RSV severity, variability in the timing of viral load quantification, and the complex interplay of host immune responses. In addition, the RSV viral load has also been associated with post-RSV wheezy episodes; however, longitudinal data beyond 3 years of age about this observation are ongoing [49].
5. Genetic susceptibility and innate immune system: Polymorphisms in immune-related genes have been suggested to influence RSV disease severity; for example, variations in IL-8 are related to more prominent inflammatory responses and the increased prevalence of wheezing following severe RSV bronchiolitis, independent of atopy [51]. Additionally, studies have shown that the innate immune system is involved in RSV progression and plays a critical role in the clinical course of RSV infections [52,53].

In addition to established epidemiological and clinical risk factors of severe RSV infection, several genetic studies have correlated host genetic factors with susceptibility to RSV-induced severe respiratory disease. A study involving whole-exome sequencing of 54 hospitalized patients with severe RSV bronchiolitis identified genetic variants, notably in olfactory and taste receptor genes, associated with susceptibility to severe RSV bronchiolitis. They identified SNP rs199665292 in the OR13C5 olfactory receptor gene as a significant candidate variant (*p*-value = 1.16 × 10^−12^; OR = 5.56). The study also highlighted HLA genes (HLA-DQA1, HLA-DPB1) and MUC4 as potential susceptibility loci. These findings indicate that genetic factors could influence RSV infection, notably implicating olfactory and taste receptors [54].

### 4.4. Mode of Transmission

The most common mode of RSV transmission is horizontal, occurring via direct contact of the nasopharyngeal or conjunctival mucosa of infected patients, or indirectly through fomite transmission, involving contaminated surfaces or objects [55,56]. Furthermore, RSV can be transmitted via respiratory droplets, which are categorized by size into large and fine (smaller than 5 µm). Fine respiratory droplets, commonly known as aerosol droplets, have the capacity for airborne transmission, potentially enabling viral deposition in the distal alveoli and contributing to more severe disease pathogenesis. The exact particle size threshold used to differentiate between droplets and aerosols has yet to be agreed upon. While a definitive particle size threshold distinguishing between droplets and aerosols remains a subject of ongoing research, the World Health Organization (WHO) and the Centers for Disease Control and Prevention (CDC) currently define transmission through particles greater than 5 µm as droplet transmission and transmission through particles 5 µm or less as aerosol transmission [55,56].

Several experimental and clinical studies proved the RSV seropositivity among infants born to women with an elevated titer of antibodies against RSV or had proved RSV infection in the third trimester. These data suggest that RSV could be transmitted vertically and may cause pulmonary and extrapulmonary disease. Further research needs to be conducted before proving this mode of RSV transmission, and further precautions should be implemented thereafter [57].

## 5. Pathogenesis

RSV is a leading cause of pediatric acute LRTIs, with a multifaceted pathogenesis involving viral entry, replication, host immune responses, and potential long-term respiratory consequences.

### 5.1. Viral Structure and Entry

RSV is an enveloped virus with a negative-sense, single-stranded, non-segmented RNA genome. It belongs to the family *Pneumoviridae*, subfamily *Pneumovirinae*, and genus *Orthopneumovirus* [58]. The virus mainly invades the ciliated epithelial cells of the respiratory tract. The viral use of host cells depends on the attachment of G glycoprotein for viral adherence to the cellular membrane and on the fusion of F glycoprotein, which mediates membrane fusion to allow viral RNA entry into the host cell cytoplasm [58,59,60]. This fusion process results in the formation of syncytia, multinucleated giant cells that contribute to airway obstruction and tissue damage [61,62].

### 5.2. Viral Replication and Spread

Once inside the host cell, RSV replicates within the cytoplasm, producing viral RNA and proteins that assemble into new virions. The formation of syncytia enables direct cell-to-cell spread of the virus, facilitating rapid dissemination within the respiratory epithelium and exacerbating tissue damage [63,64].

### 5.3. Host Immune Response

The host’s immune response to RSV involves both innate and adaptive components. As per innate immunity, the virus is detected by pattern recognition receptors, which, upon activation, produce interferons and pro-inflammatory cytokines. When activated in excess, this response recruits immune cells to the site of infection but can also result in tissue damage [62,65,66]. However, the clearance of RSV infection depends critically on T cells, which are an important element of the adaptive immune response [66]. Notably, RSV may influence the immune response in such a way that immunological memory may be insufficient, and it is very possible for reinfection to occur [14,64]. Several studies highlight the specific signalling pathways involved in host immune responses to RSV, such as the role of type I interferon (IFN), T-cell subsets, and dendritic cells. With regard to the IFN, RSV suppresses the host’s innate immune response through non-structural proteins (NS), NS1 and NS2, which are effective at inhibiting IFN signaling. Specifically, RSV-NS1 interferes with the IFN-α Janus Kinase/Signal Transducers and Activators of Transcription (JAK/STAT) pathway by blocking STAT1 nuclear translocation, a key protein in the JAK-STAT signaling pathway, which is crucial for antiviral responses [67]. While the innate immune response plays an important role in defense against RSV infection, RSV has developed effective strategies to manipulate the host’s innate immune responses through NS1 and NS2 [68].

### 5.4. Immunopathogenesis

RSV infection severity is governed by the balance between viral replication and host immune reactions. Airway passage inflammation, mucus production, epithelial cell destruction, airway obstruction, and impaired gas exchange can all be attributed to excessive immune activation. Infants, being immunologically immature, are known to be more susceptible to severe RSV disease [62,69].

### 5.5. Genetic Variability and Immune Evasion

The G protein of RSV has been confirmed as a genetically variable element in many studies and is largely responsible for RSV evasion from host immune responses. The significant amount of antigenic diversity has led to difficulty in developing a vaccine against RSV infection; furthermore, reinfection can occur repeatedly over the course of one’s life [64,70].

### 5.6. Pleiotropic Effects

Beyond the primary acute respiratory manifestations of RSV, affecting both the upper and lower respiratory tracts, several studies have demonstrated chronic airway involvement and extrapulmonary manifestations, which are known as pleiotropic effects. A recently published review article summarized the neurological effect of RSV infection, particularly vertically transmitted infection, which includes a spectrum of neurological manifestations such as seizures, neonatal encephalopathies, and dysphagia [57]. Furthermore, Eisenhut et al. [71] showed, in a systematic review, that RSV infection can involve cardiac tissues, manifesting as myocarditis, heart failure, and cardiac arrhythmias, including supraventricular and ventricular tachycardia. Overall, these reviews clearly indicate that there are significant extrapulmonary manifestations of RSV and should be considered in treating patients with RSV infection.

## 6. Diagnosis

The timely and accurate diagnosis of RSV infection in children is clinically imperative and critically important for effective infection control. Clinical evaluations and various laboratory techniques constitute rational and practical diagnostic approaches, each with its own advantages and limitations. Although national and international guidelines concur that the diagnosis of RSV infection is primarily clinical, the etiological confirmation through viral isolation remains crucial [5,22]. The unwarranted escalation of antimicrobial therapy in the absence of definitive RSV testing represents a prevalent clinical practice [72]. Furthermore, the mandated implementation of RSV prophylaxis necessitates widespread diagnostic testing to accurately evaluate the effectiveness of monoclonal antibodies and to determine the optimal timing for their initiation [73]. Further studies, which take variability in healthcare resources worldwide into account, will need to be undertaken.

### 6.1. Clinical Assessment

RSV infection is classically characterized by nonspecific respiratory symptoms such as rhinorrhea, cough, and wheezing, which sometimes progresses to bronchiolitis or pneumonia in more protracted cases. However, these manifestations can overlap with the clinical presentations of other respiratory viral infections, making clinical diagnosis even more difficult. Consequently, while clinical assessment is vital, confirmation in the laboratory is frequently required for a definitive diagnosis [74]. RSV-induced acute bronchiolitis, a common lower respiratory tract infection in infants and young children, frequently requires objective clinical assessment to guide appropriate therapeutic interventions. Several scoring systems have been developed to standardize the assessment of bronchiolitis severity, incorporating clinical parameters, such as respiratory rate, oxygen saturation, wheezing, retractions, and overall appearance. Among the most widely utilized tools are the Wang Bronchiolitis Severity Score (WBSS), Kristjansson Respiratory Score (KRS), and Global Respiratory Severity Score (GRSS) [75]. Each system differs slightly in the parameters assessed, the scoring criteria, and the age range it is designed for. For example, while the WBSS focuses on observable symptoms like retractions and audible wheezing, the KRS incorporates the degree of respiratory compromise, and the GRSS extends the evaluation to include oxygen saturation and lethargy. By comparing these scoring systems, clinicians can gain insight into their similarities, differences, and applicability in various clinical settings [76].

### 6.2. Laboratory Diagnostic Methods

Several laboratory techniques have been employed to confirm RSV infections:

- Rapid Antigen Detection Tests (RADTs): These tests detect RSV antigens in respiratory specimens and provide rapid results that may be of immediate clinical value. However, the sensitivity of these tests differs depending on several practical and logistic factors. Specifically, RADTs have demonstrated greater sensitivity in young children, who typically shed more virus, and less sensitivity in older children and adults [77,78].
- Nucleic Acid Amplification Tests (NAATs): These tests are described as highly sensitive and specific for the detection of RSV RNA. A number of RNA detection techniques have been employed, e.g., reverse transcription–polymerase chain reaction (RT-PCR). NAATs are recognized as useful across the age spectrum of patients and can detect low viral load counts in both clinical and research settings [79,80].
- Virus culture: The cell culture isolation of RSV, although previously considered the gold standard for identifying the virus, is now recognized as time-consuming and, hence, is less commonly used as a routine diagnostic method [81].

Onwuchekwa et al. (2023) conducted a systematic review and meta-analysis comparing the performance of multiple RSV diagnostic modalities in children [82]. Compared to RT-PCR, the gold-standard diagnostic tool, they concluded that direct fluorescent antibody tests, viral culture, and RADTs had respective sensitivities of 87%, 76%, and 74%. The pooled specificity of these tests was ≥98%, indicative of high accuracy in RSV confirmation [82].

The sensitivity of RSV detection has also been proven to be significantly affected by the choice of specimen. Specimens commonly used for RSV confirmation include the following:

- Nasopharyngeal swabs: Widely used due to the ease of collection and they mostly yield good sensitivity results.
- Nasopharyngeal aspirates: More sensitive than swabs but also more invasive; considered the gold standard for RSV detection.
- Nasal swabs: Less invasive and more comfortable, useful in outpatient settings.

Findings from the same systematic review by Onwuchekwa et al. (2023) indicate that the addition of other specimen types to NPS/NPA RT-PCR testing yields only a marginal increase in RSV detection rates (1–10% on average) [82]. This suggests that routine testing using a single NPS or NPA specimen in children presenting with lower respiratory tract infection is unlikely to miss a substantial proportion of RSV cases [82].

### 6.3. Differential Diagnosis

Differentiating RSV from other respiratory pathogens is necessary, as influenza, parainfluenza, and SARS-CoV-2 may also produce similar clinical features. Experiences in many clinical settings worldwide indicate that differentiating between infections with these viruses requires comprehensive diagnostic panels and careful clinical evaluations or molecular typing [74,83,84].

### 6.4. Advances in RSV Diagnostic Approaches

Several recent advancements have enhanced the rapidity and accuracy of RSV diagnosis:

- Point-of-Care Molecular Assays: The rapid and accurate detection of RSV at one’s bedside using these assays has shown great potential to provide timely clinical decision making and appropriate patient management [82,85,86].
- Multiplex PCR Panels: These panels can simultaneously detect multiple respiratory pathogens, including RSV, thereby facilitating the identification of co-infections and offering guidance for targeted therapies [82,85,87].
- Biomarkers: Biomarker research may not only help differentiate RSV from other viral infections but also predict severity and guide treatment strategies [74].

Incorporating these sophisticated diagnostic devices into clinical practice may lead to better management outcomes for RSV in children.

## 7. Management

RSV infection in children is managed through supportive care, pharmacological interventions, and prevention. The approach used depends on the severity of the disease, the age of the patient, and their general health.

### 7.1. Supportive Care

RSV infection treatment continues to be primarily supportive in nature, involving treating symptoms and maintaining adequate oxygenation and hydration. Key components of such care include the following:

- Oxygen therapy: This is given to children with hypoxemia (who have an oxygen saturation of less than 90%).
- Hydration: Sufficient fluid intake must be maintained to prevent dehydration, which may sometimes require intravenous fluids in severe cases.
- Nutritional support: Ensuring adequate nutrition is crucial, especially for infants experiencing feeding difficulties because of respiratory distress.

The details of these classic supportive care measures for RSV infections have been adequately summarized in several previously published articles [22,88,89,90,91]. For managing mild to moderate RSV infections and preventing progression to severe disease, all of these measures appear to be imperative and crucial.

### 7.2. Pharmacological Interventions

The pharmacological treatments for RSV are limited, with current options including the following:

- Antiviral agents: Many clinical trials have confirmed that the antiviral drug ribavirin has limited benefits; moreover, it is recommended for use solely in high-risk and severe cases because of the associated adverse effects [20,90,91,92,93]. However, in the absence of other effective antiviral agents, it is also considered a reasonable option for treating RSV patients with hematologic malignancy or who are recipients of haematopoietic stem cell transplants [94]. Therefore, addressing the urgent need for effective therapeutics targeting RSV must be recognized as a top global public health priority.

## 8. Prevention

The prevention of RSV infections is crucial, as it will preclude the resultant morbidity, mortality, and substantial economic burden. The highest risk of severe disease is found among infants, especially those younger than 6 months, and among children with pre-existing health conditions [43]. Innovative immunoprophylaxis, maternal vaccination, and public health strategies have been used to prevent RSV infections. Nevertheless, addressing barriers of access, cost, and coverage in vulnerable populations remains an urgent concern [22,30,90]. Moreover, due to the high prevalence of RSV reinfection as well as the lack of long-lasting RSV protection, further effective pediatric vaccines are urgently needed.

### 8.1. Immunoprophylaxis

Immunoprophylaxis using monoclonal antibodies has been a cornerstone in preventing RSV and provides immediate passive immunity to high-risk infants. However, a recently introduced long-acting monoclonal antibody promises to greatly change RSV prevention strategies [95,96,97,98].

#### 8.1.1. Palivizumab

The monoclonal antibody palivizumab, which targets the F protein of RSV, has demonstrated great efficacy in reducing the rate of hospitalization due to RSV in high-risk infants, including those born premature or those with congenital heart and lung diseases [99,100]. However, the monthly intramuscular administration of this antibody throughout the RSV season is deemed impractical, and its use in low-resource settings is not financially tolerable [101,102]. Thus, while palivizumab represents a viable option in high-income countries, cost-effective alternatives are still needed.

#### 8.1.2. Nirsevimab

The recently approved nirsevimab represents a major step forward in RSV immunoprophylaxis. It is a long-acting monoclonal antibody, for which only one intramuscular injection is needed to offer season-long protection [89]. Based on results from clinical trials, nirsevimab reduces the risk of medically attended RSV-associated LRTIs by 74.5% and the risk of hospitalization by 62.1% in healthy late-preterm and term infants [28].

The efficacy of single-dose nirsevimab and its improved affordability make it a highly promising candidate for increased implementation in both high- and low-income countries. Its approval in many European countries and the United States represents a paradigm shift in RSV prevention, with promise for global impact and the achievement of equitable access [102,103,104,105].

#### 8.1.3. Clesrovimab and Next-Generation Antibodies

Ongoing research into next-generation monoclonal antibodies is progressing toward new and promising immunoprophylactic agents. Clesrovimab, an anti-idiotypic monoclonal antibody designed to bind to the RSV F protein, was recently released and has been demonstrated to be more efficacious, durable, and affordable compared to previously applied antibodies [106].

Collectively, next-generation RSV antibodies are directed to target conserved epitopes on the F protein to minimize resistance development and maximize the breadth of protection. Innovations in antibody engineering aimed at extending half-life have the potential to further simplify RSV prevention [107].

### 8.2. Vaccination

Vaccination remains the ultimate goal in RSV prevention. Active immunization strategies seek to engender long-term immunity to RSV, thereby reducing disease severity and complications. Maternal and adult vaccine development is driving significant evolution in RSV prevention strategies.

#### 8.2.1. Maternal Vaccination

Maternal immunization during pregnancy has been confirmed as an important strategy to protect newborns, who are at the highest risk of severe RSV disease. Recent advancements in RSV prevention include the approval of two vaccines by the US Food and Drug Administration for use in adults over 60: Abrysvo (Pfizer) and Arexvy (GSK). Notably, Abrysvo has also received FDA approval for administration to pregnant individuals between 32 and 36 weeks of gestation. Pfizer’s RSVpreF vaccine, which targets the viral F protein, has demonstrated promising results in clinical trials. Specifically, the MATISSE trial reported that maternal vaccination significantly reduced the incidence of severe RSV disease in infants, by up to 82% within the first 3 months of life and by 70.9% through the first 6 months [108].

Maternal vaccination is beneficial in high-income country and low-resource settings, where access to healthcare is limited, providing passive immunity during the early critical months of life and substantially reducing mortality among newborns and infants [109,110,111,112].

#### 8.2.2. Pediatric Vaccination

Using a formalin-inactivated vaccine technique, the first RSV vaccine was made in the 1960s. The vaccine recipients had good a antibody response but were later observed to have vaccine-enhanced respiratory disease (ERD) and needed hospitalization [25]. Two infants developed fatal pulmonary disease, one at age 14 months, the other at age 16 months. The proposed mechanisms of the ERD were a high level of antibodies but poorly neutralizing and the abnormal immune response by the vaccine that led to a T-helper 2-biased response [25]. Since then, several RSV vaccines in late-stage development have been designed for direct administration to infants and young children [7]. The majority of these vaccines primarily target the pre-fusion conformation of the F glycoprotein, which is involved in viral attachment and fusion to the host cell [98]. These vaccines have demonstrated strong immunogenicity and safety profiles in early clinical data, and more exploratory studies have investigated long-term efficacy and optimal vaccination schedules. Interest is also increasing in the intranasal administration of live attenuated vaccines to exaggerate the positive characteristics of human immunity and induce both systemic and mucosal immunity. Meanwhile, subunit vaccines and nanoparticle-based vaccines show promise to potentially enhance immunogenicity in children with immature immune systems [107,112]. A mRNA vaccine, which is assumed to represent a more successful and effective preventive technology, has also been discussed and attempted [101]. To date, 24 vaccines are under development against RSV for pediatric and old adult populations, and 2 of them are licensed for market use [113,114].

## 9. Prognosis and Long-Term Outcomes

The effects of RSV infection in children vary from mild upper respiratory symptoms to severe LRTIs requiring hospitalization and intensive care. Although most children recover uneventfully, increasing recognition should be given to the repercussions of severe infections in relation to childhood quality of life, respiratory health, and socioeconomic outcomes. The importance of long-term follow-up and early intervention strategies for at-risk children is highlighted by a growing body of evidence [10].

### 9.1. Acute and Critical Infection Outcomes

RSV continues to be the most common cause of LRTIs in children under 5 years of age and is associated with substantial morbidity and mortality [115,116,117]. In 2015, RSV-associated LRTIs were estimated to have caused 33.1 million episodes of infection, 3.2 million hospitalizations, and 59,600 in-hospital deaths in children <5 years worldwide [90]. The burden of this disease is greatest in infants less than 6 months of age, with preterm infants and those having underlying conditions such as congenital heart disease, chronic lung disease, or immunodeficiency at higher risk of severe disease, requiring intensive medical support [10,23].

In the clinical course, RSV infection is linked to bronchiolitis and pneumonia and is often associated with hypoxia, respiratory failure, and prolonged hospitalization. Accordingly, early mortality and short-term morbidity are crucially dependent on the anticipation and prompt recognition of complications.

### 9.2. Long-Term Respiratory Complications

Infants who required mechanical ventilation for RSV infections typically have chronic respiratory complications that persist, sometimes into childhood and even into adolescence [9]. Severe RSV infections among infants are associated with extended hospital stays and a higher risk of chronic respiratory disorders, such as asthma and recurrent wheezing [114,118].

#### 9.2.1. Recurrent Wheezing and Asthma

Infants who have acute RSV bronchiolitis are much more likely to develop recurrent wheezing and asthma. A comprehensive systematic review by Fauroux et al. (2017) found that having RSV bronchiolitis before the age of 5 increased the risk of wheezing in the following year of life [10]. In addition, genetically susceptible children who experience RSV-induced airway inflammation may experience persistent hyperreactivity in the airways, as there is evidence of the long-term remodelling of airway structures [10]. RSV bronchiolitis may trigger a Th2-skewed immune response, leading to airway eosinophilia and increased susceptibility to asthma [118]. New research outcomes also suggest that RSV-mediated immune dysregulation and its association with environmental exposures such as tobacco smoke and air pollutants make chronic respiratory sequelae more likely to occur [10,17].

#### 9.2.2. Impaired Lung Function

Several longitudinal studies have confirmed that children who experienced more severe RSV infections tend to have lower lung function parameters, measured as forced expiratory volume, compared to age-matched healthy controls [10]. This is because RSV infection may result in persistent structural and functional effects on developing airways, thus impacting lung function, which often becomes evident in adolescence or early adulthood. Even more pronounced pulmonary dysfunction has been observed in infants mechanically ventilated due to RSV bronchiolitis [119].

## 10. Implications for Clinical Practice

Because of the devastating consequences of severe RSV infections, children who recover need active and multidisciplinary management by healthcare providers. The early identification of chronic respiratory sequelae is critical, as early intervention can prevent further morbidity. Children at high risk should regularly undergo pulmonary function testing, be screened for asthma symptoms, and follow a regimen that includes inhaled corticosteroids and bronchodilator therapy for those with the asthma phenotype [10,120].

Maternal RSV vaccination and monoclonal antibody prophylaxis, including nirsevimab, must be integrated into clinical practice to prevent severe disease in infants. Furthermore, healthcare systems should focus on family-centred care to deal with the psychosocial and financial challenges faced by caregivers.

## 11. Future Directions

Much of the future research concerning RSV should focus on understanding the mechanistic links between RSV infection and long-term respiratory outcomes, especially the development of asthma and impaired lung function. The high morbidity rates associated with severe RSV and chronic sequelae may be prevented by the early detection of at-risk children through advances in biomarkers and the development of targeted therapies [84]. Ongoing antiviral agent and immunomodulator trials also have the potential to ameliorate acute disease severity and limit long-term respiratory damage [10,23,52].

In addition, addressing disparities related to RSV care necessitates global collaboration. Reducing global RSV-related mortality and long-term morbidity depends on improving access to vaccines, affordable monoclonal antibodies, and robust healthcare infrastructure in resource-limited settings.

## 12. Research Gaps and Future Directions

RSV infections are manageable, but significant gaps in our understanding require more focused research. Improving knowledge of RSV epidemiology, developing effective and accessible preventive strategies, improving disease therapy options, and investigating long-term health outcomes for children affected by RSV will provide avenues for better addressing this challenging virus.

### 12.1. Gaps in RSV Epidemiology

Historically, RSV typically follows a seasonal cycle, but the COVID-19 pandemic has disrupted those patterns, creating an urgent need to comprehend the changes in RSV transmission dynamics. Important gaps in our understanding of post-pandemic RSV epidemiology are highlighted by shifts in seasonality, surges in infections following public health restrictions, and changes in the age distribution of RSV cases [82,121]. To monitor RSV trends and guide public health interventions, enhanced global surveillance systems are needed, particularly in LMICs. Many LMICs underreport and have a limited understanding of the true burden of RSV in their countries due to insufficient surveillance infrastructure. The use of real-time RSV surveillance can lead to improved early detection, more rapid responses to outbreaks, and the wider deployment of vaccines worldwide. In Saudi Arabia, there is an urgent need for comprehensive epidemiological studies and clinical trials focusing on RSV infections. Research should aim to evaluate the effectiveness of preventive strategies, including maternal vaccination and monoclonal antibodies, tailored to the region’s specific healthcare context.

### 12.2. Gaps in RSV Vaccine Development and Preventive Strategies

Maternal RSV vaccines, such as the recently approved RSVpreF and monoclonal antibodies like nirsevimab, provided recent improvements in RSV prevention, but gaps remain in our knowledge of how these interventions work in the long term for diverse populations, including efficacy, safety, and cost-effectiveness. More research is needed to fully understand their performance in areas where RSV incidence is highest, particularly in LMICs [64]. Furthermore, the feasibility of integrating RSV vaccines into existing immunization programmes and thereby enhancing population access and coverage should be studied. Key priorities include the development of cost-effective vaccination strategies for pediatric populations and, most importantly, monoclonal antibody therapies, such as single-dose monoclonal antibodies for high-risk infants.

### 12.3. Gaps in Treatment Options

There are currently very few effective therapeutic interventions for RSV, even for cases of severe disease. Supportive care has been the treatment of choice, and there remains an urgent need for the development of safe and effective antivirals to limit RSV-related morbidity. Existing options, such as ribavirin, are not commonly used due to their limited effectiveness and potential toxicity. The current emerging research into novel antiviral candidates and immunotherapies, which can be evaluated for safety and efficacy on children with severe RSV infection, needs to be accelerated through clinical trials [17,27,28,62,64]. Furthermore, studies are needed to elucidate dosing schedules, resistance patterns, and the real-world effectiveness of monoclonal antibodies for preventing severe RSV in high-risk groups.

## 13. Future Directions and Emerging Technologies

A number of new technologies hold promise for improving RSV prevention and management. Mathematical modelling can help predict RSV seasonality and estimate the potential impact of vaccination and immunoprophylaxis programmes at the population level [23,28,62]. Personalized medicine approaches based on genomic and immunologic data may be able to identify children at higher risk of severe disease for targeted interventions. The identification of biomarkers indicative of disease severity and treatment response could change clinical management and lead to better outcomes for RSV-infected children. Most of all, an important aspect of overcoming RSV challenges worldwide is collaboration between researchers and governmental and healthcare organizations at the global level. Large-scale clinical trials, data sharing, and the adoption of evidence-based interventions that take populations into account can all be supported by international partnerships. Ultimately, closing these gaps will help us better address the burden of RSV on children, improve children’s long-term health, and narrow global health disparities.

## 14. Conclusions

RSV is one of the most important pathogens causing acute LRTIs in infants and young children throughout the world. Most RSV infections are mild, but severe disease among vulnerable populations, including premature infants, children with chronic comorbidities, and resource-limited settings, remains responsible for substantial morbidity, hospitalizations, and mortality worldwide. Disruptions in RSV seasonality resulting from the COVID-19 pandemic, recent epidemiological evolution of the RSV, and its emerging epidemiology all highlight the importance of a robust surveillance system and associated public health strategies.

Rapid progress has been made in the area of RSV prevention, including the development of maternal vaccines such as RSVpreF and long-acting monoclonal antibodies like palivizumab and nirsevimab, which promise to significantly reduce the disease burden. However, equitable access to these interventions remains challenging, with gaps persisting, largely in LMICs with the highest burden. Given the limited availability of effective antiviral therapies, continued investment in both novel treatments and clinical research to optimize their use in pediatric populations is urgently needed.

Long-term respiratory sequelae, including recurrent wheezing, asthma, and impaired lung function, have been linked to severe RSV infection in the first year of life. Mitigating the global impact of RSV necessitates not only enhancing education, improving access to healthcare, and implementing tailored public health initiatives but also addressing environmental and socioeconomic disparities.

Finally, minimal advances have been made in understanding, preventing, and managing RSV infections, with more needed to bridge extant research gaps. Enhancing surveillance systems, increasing equitable access to vaccines and therapeutics, and conducting scientific research to elucidate RSV pathogenesis and long-term outcomes will all be important for reducing RSV-related morbidity and mortality. Focusing on these strategies will enable us to reduce the RSV burden worldwide, improve patient outcomes, and move closer to eliminating RSV as a major public health threat.
